# Fractalkine modulates pulmonary angiogenesis and tube formation by modulating CX3CR1 and growth factors in PVECs

**DOI:** 10.1515/biol-2022-0670

**Published:** 2023-12-31

**Authors:** Jun Liao, Xianwu Yang, Jiejie Yang, Jingjing Xiao, Xuyang Liu, Yingquan Zhuo, Jiafei Yang, Huajian Gu

**Affiliations:** Department of Pediatric Surgery, Affiliated Hospital of Guizhou Medical University, No. 28, Guiyi Street, Yunyan District, Guiyang 550002, P. R. China; Department of Hepatobiliary Surgery, Affiliated Hospital of Guizhou Medical University, Guiyang, P. R. China

**Keywords:** fractalkine, CX3CR1, hepatopulmonary syndrome, tube formation, macrophages

## Abstract

This study aimed to investigate effects of pulmonary fractalkine (FKN/CX3CL1) on angiogenesis and tube formation. Tube forming capability of pulmonary vascular endothelial cells (PVECs) was evaluated. CCK-8 assay was used to evaluate proliferation of PVECs. RT-PCR assay was used to determine angiogenesis specific biomarkers. Western blot was applied to identify CX3CR1, Akt, phosphorylated Akt (p-Akt), Erk1/2, phosphorylated Erk1/2 (p-Erk1/2), vascular endothelial growth factor A (VEGFA), and inducible nitric oxide synthase (iNOS) expression. VEGF-A and platelet-derived growth factor (PDGF) levels were examined using ELISA. FKN was safe and triggered tube formation in PVECs. FKN significantly enhanced VEGF-A, PDGF, and iNOS gene transcription compared to the Control group (*p* < 0.05). CX3CR1 interfering (LV5-CX3CR1 shRNA) remarkably reduced CX3CR1 expression compared to those in LV5 blank group (*p* < 0.05). Ratios of p-Akt/Akt and p-Erk/Erk were significantly decreased in CX3CR1 shRNA-treated PVECs administered Akt inhibitor (or Erk inhibitor) and 10 ng/mL FKN compared to CX3CR1 shRNA-treated PVECs administered 10 ng/mL FKN (*p* < 0.05). FKN increased VEGF-A and iNOS expression through activating Akt/Erk pathway. FKN promoted VEGF-A/iNOS expression and triggered p-Akt/Akt and p-Erk/Erk pathway through modulating CX3CR1. FKN-treated macrophages enhanced activation of Akt/Erk pathway. FKN-treated macrophages enhanced PDGF and VEGF-1 expression in PVECs. FKN modulated pulmonary angiogenesis and tube formation through modulating CX3CR1 and growth factors and activating p-Akt/Akt and p-Erk/Erk signaling pathway.

## 1 Introduction

Hepatopulmonary syndrome (HPS) is a progressive disease characterized by worsening hypoxemia due to intrapulmonary vascular dilatation, arteriovenous malformations, and increased vasoactive substances in the chronic liver disease [1,2]. Prevalence of HPS varies from 4 to 47% due to different cut-offs in defining arterial hypoxemia in primary studies, and mortality rate of HPS is about 41% [3]. Over the past two decades, the pathogenesis and precise mechanisms of HPS were under active investigation. Based on the experimental and clinical research, the mechanisms of HPS continue to be uncovered, which thus provides the chance of clearly understanding the HPS pathogenesis and potential therapeutic targets [4]. Although progress has been made in delineating the mechanisms underlying the imbalance of vasoactive substances, pulmonary vascular alterations, and angiogenesis in HPS, to date, there is still lack of related effective therapeutic approaches apart from liver transplantation [5].

Fractalkine (FKN or CX3CL1), as a member for CX3C molecule family, plays critical roles for initiating and developing the inflammation [6]. The FKN/CX3CL1 could bind to CX3C chemokine receptor 1 (CX3CR1) and trigger the pro-inflammatory responses [7]. FKN/CX3CL1 usually expresses on a series of cells, including dentritic cells, endothelial cells (ECs), and the intestinal ECs [8,9]. Recently, a few documents [10,11] reported that interaction between the FKN/CX3CL1 and the CX3CR1 is correlated with the angiogenesis and pathogenesis for the atherosclerosis. Meanwhile, FKN/CX3CL1 might also involve in the angiogenic processes via activating the endothelial cells [12]. We speculated that FKN/CX3CL1 might play critical effects on the pathogenesis of the HPS. Therefore, this study investigated the effects of FKN/CX3CL1 on pathogenesis of HPS and explored the underlying molecular pathological mechanisms.

## 2 Materials and methods

### 2.1 Cell culture, treatment, and grouping

The mouse pulmonary vascular endothelial cells (PVECs) were purchased from ATCC cell bank (Manassas, VA, USA) and cultured in Dulbecco’s modified Eagle medium (Gibco BRL Co. Ltd, Grand Island, NY, USA) supplemented with 10% fetal bovine serum (FBS, Gibco BRL), and containing penicillin (100 U/mL, Beyotime Biotech, Shanghai, China) and streptomycin (100 mg/mL, Beyotime Biotech). The PVECs were cultured and maintained in the humidified incubator at 37℃ under condition of 5% CO_2_.

PVECs in the Control group were administered the synthesized CX3CR1 shRNA only. PVECs in 10 ng/mL FKN group were administered the synthesized CX3CR1 shRNA and 10 ng/mL FKN. PVECs in Akt inhibitor + 10 ng/mL FKN group were administered the synthesized CX3CR1 shRNA, 10 ng/mL FKN, and 5 μM of Akt inhibitor (AZD5363). PVECs in Erk inhibitor + 10 ng/mL FKN group were administered the synthesized CX3CR1 shRNA, 10 ng/mL FKN, and 30 μM of Erk inhibitor (PD98059). Both Akt inhibitor and Erk inhibitor were administered simultaneously, and incubated for 30 min. Furthermore, the proliferation of the PVECs was evaluated using the cell counting kit 8 (CCK-8) assay according to the protocol of the manufacturer (Cat. No. C0037; Beyotime Biotech, Shanghai, China).

### 2.2 Tube formation assay *in vitro* levels

The tube forming capability of the PVECs (the tubule-like structures) was evaluated with the tube formation assay as described by a previous study [13], with a few modifications. In brief, the 24-well plates were pre-treated using the ice-cold Matrigel solution (BD biosciences, Franklin Lakes, NJ, USA) and then incubated for another 30 min at 37℃ to make Matrigel to solidify. The PVECs were cultured, harvested, and suspended in the FBS containing medium (2% FBS), and cultured in the Matrigel-pre-treated wells with density of 10 × 10^4^ cells/well at 37℃ for 30 min. Finally, the tubule-like structures were assigned as the formed tubes.

### 2.3 Real time PCR (RT-PCR) assay

The angiogenesis specific biomarkers, including vascular endothelial growth factor A (VEGFA), inducible nitric oxide synthase (iNOS), and platelet-derived growth factor (PDGF), were detected using the RT-PCR assay. Briefly, the total RNAs were isolated from the PVECs undergoing different treatments, using the commercial RNeasy Mini Kit (Cat. No. 74104; Qiagen, Hilden, Germany) as instructed by protocol of manufacturer. Then, a total of 0.5 μg of RNA was synthesized to the complementary DNA with PrimerScript RT Reagent Kit (Cat No. RR037A; Takara Bio., Dalian, China) as described by the manufacturer. The gene transcriptions of VEGFA, iNOS, and PDGF were determined with the Sybr Premix-Ex Taq (Cat No. DRR420A; Takara Bio., Dalian, China) on the ABI Real-time PCR Amplifying System (Model: PRISM 7500, ABI, Foster City, CA, USA). The primers are listed in Table 1. The RT-PCR amplifying conditions are listed as the follows: 95℃ for 4 min, followed by 35 cycles at 95℃ for 10 s and 60℃ for 30 s. The relative gene transcriptions of the above genes were analyzed with the 2^−ΔΔCT^ method [14].

**Table 1:** Primers for the RT-PCR assay

Genes		Sequences (5′–3′)	Length (bp)
VEGF	Forward	ATCATGCGGATCAAACCTCAC	96
	Reverse	TGTTCTGTCTTTCTTTGGTCTGC	
PDGF	Forward	CGCACCAACGCCAACTTC	154
	Reverse	TGGGCTTCTTTCGCACAATC	
iNOS	Forward	TTGGAGCGAGTTGTGGATTG	147
	Reverse	GGTCGTAATGTCCAGGAAGTAGG	
	Reverse	ATGTCACGCACGATTTCCC	

### 2.4 Western blot assay

The cellular proteins in the PVECs were extracted using the ice pre-treated radioimmunoprecipitation assay (Beyotime Biotech) containing the protease inhibitor. The protein’s concentration was examined with a commercial BCA detection kit (Cat. No. P0010S; Beyotime Biotech) as instructed by the protocol of the manufacturer. Then, a total of 20 μg of protein in each group was separated with the 15% SDS-PAGE and electronically transferred onto the PVDF membranes (Beyotime Biotech). Post blocking with the non-fat milk (5%) dissolving in the PBS containing 0.1% Tween-20 for 60 min at 25℃, the PVDF membranes were incubated overnight at 4℃ with the rabbit anti-mouse CX3CR1 polyclonal antibody (Cat. No. ab8021, 1:1,000), rabbit anti-mouse Akt polyclonal antibody (Cat No. ab8805, 1:1,000), rabbit anti-mouse phosphorylated Akt (p-Akt, phospho T308), polyclonal antibody (Cat. No. ab38449, 1:1,000), rabbit anti-mouse Erk1/2 monoclonal antibody (Cat No. ab184699, 1:1,000), rabbit anti-mouse phosphorylated Erk1/2 (p-Erk1/2, phospho T202 + Y204), polyclonal antibody (Cat No. ab200807, 1:1,000), rabbit anti-mouse VEGFA polyclonal antibody (Cat No. ab51745, 1:1,000), rabbit anti-mouse iNOS monoclonal antibody (Cat No. ab115819, 1:1,000), and rabbit anti-mouse β-actin monoclonal antibody (Cat No. ab8226, 1:1,000) overnight at 4℃. Then, the PVDF membranes were washed using PBST three times (5 min per time) and incubated using the horse radish peroxidase-conjugated IgG (Cat No. ab6721, 1:1,000) for 2 h at room temperature. All the above first and second antibodies were purchased from Abcam Biotech (Cambridge, MA, USA). The ECL kit was used for conducting the chemiluminescent detection for the protein expressions. The western blot bands were analyzed with the Image Pro-Plus imaging software (version: 6.0, Media Cybernetics Inc.). The relative of above molecules was represented as the ratio of the target molecule to internal control β-actin.

### 2.5 Measurement for the VEGF-A and PDGF levels

The VEGF-A and PDGF levels in the supernatants of PVECs were measured using the commercial Mouse VEGF-A ELISA Kit (Cat No. ab119566; Abcam Biotech) and Mouse PDGF ELISA Kit (Cat. No. ab224879; Abcam Biotech), as instructed by the protocols of the manufacturers.

### 2.6 Statistical analysis

The data in this study were represented as mean ± SD and analyzed using the professional GraphPad Prism software (version: 8.0, GraphPad Software, Inc., La Jolla, CA, USA). The statistical analysis was conducted with the one-way ANOVA validated by Tukey’s *post hoc* test. *P* values less than 0.05 were assigned as the significant differences.

## 3 Results

### 3.1 Fractalkine (FKN) treatment is not toxic to the PVECs

**Figure 1:**
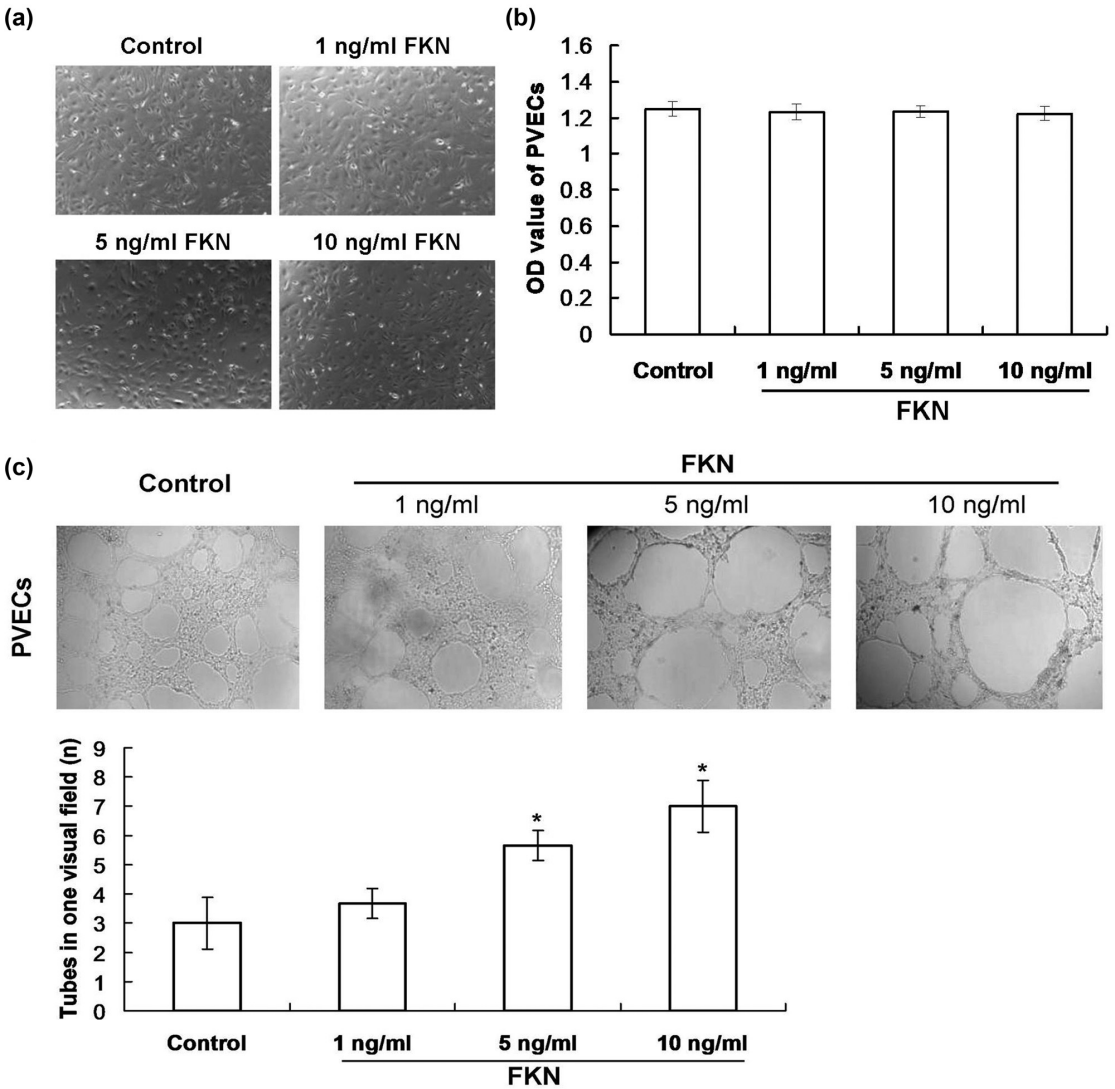
Evaluation for the effects of FKN on proliferation and tube formation of PVECs undergoing different dosages of FKN treatments. (a) Effect of FKN on proliferation. Magnification, 100×. (b) Evaluation for the OD value of the PVECs in different groups. (c) Effect of FKN on tube formation and statistical analysis. Magnification, 100×. FKN: fractalkine; PVECs: pulmonary vascular endothelial cells.

In order to clarify the toxic effects of FKN on the PVECs, we observed the proliferation status of PVECs by microscope. According to the proliferation of PVECs, we found that there was no obvious toxicity appearing in the FKN-treated PVECs (Figure 1(a)). The CCK-8 findings also showed that there were no significant differences for the optical density (OD) values of PVECs among different groups (Figure 1(b)). Therefore, the FKN (from 1 to 10 ng/mL) is safe for the proliferation of PVECs.

### 3.2 FKN treatment triggered tube formation in PVECs

We investigated the effects of the different concentrations of FKN on tubular capability of the PVECs to differentiate the tube-like structures. As demonstrated in Figure 1(b), the PVECs exposing to the FKN differentiated to form the network of the tube-like structures with a dosage-dependent manner. For the 1 ng/mL FKN treating PVECs which elongated and connected with each other to form a tube-like network (Figure 1(c)), with more tube-like structures than the Control group. The tube-like structures were obviously more in PVECs undergoing 5 ng/mL FKN (*p* < 0.05) and 10 ng/mL FKN (*p* < 0.05) compared to those of PVECs in the Control group (Figure 1(c)). This process peaked in PVECs administered with 10 ng/mL of FKN.

### 3.3 FKN treatment enhanced VEGF-A, PDGF, and iNOS gene transcription

**Figure 2:**
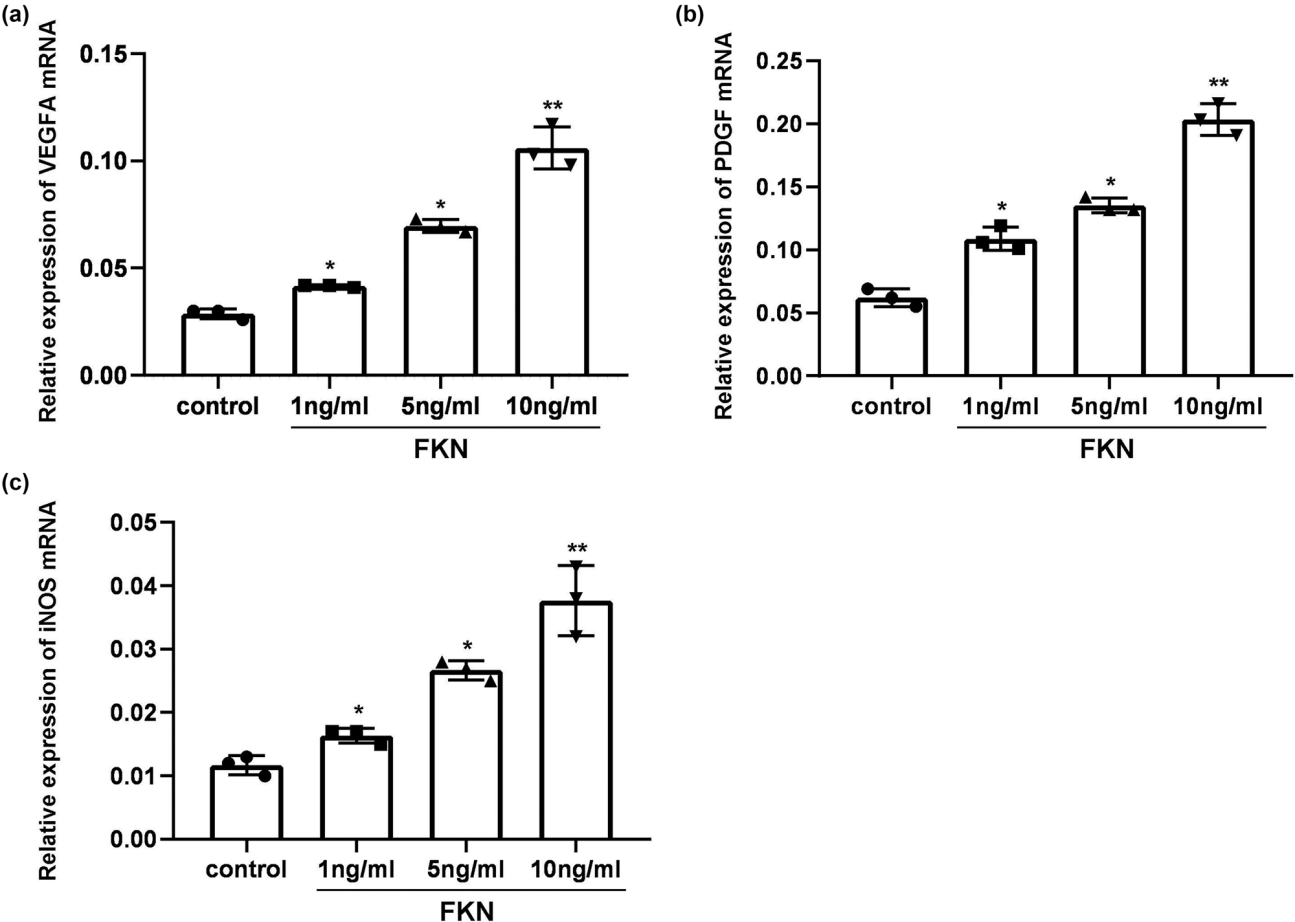
Effects of FKN treatment on gene transcription of growth factors, including VEGF-A, PDGF, and iNOS. (a) Gene transcription of VEGFA. (b) Gene transcription of PDGF. (c) Gene transcription of iNOS. *p < 0.05 versus 0 ng/mL FKN treatment. VEGFA: vascular endothelial growth factor A, iNOS: inducible nitric oxide synthase, PDGF: platelet-derived growth factor.

The growth factors, including VEGF-A, PDGF, and the downstream angiogenic signaling mediator, iNOS, were determined using RT-PCR assay. The results demonstrated that gene transcriptions of VEGF-A (Figure 2(a)), PDGF (Figure 2(b)), and iNOS (Figure 2(c)) in FKN administered PVECs were markedly higher compared to those in PVECs in Control group (*p* < 0.05). Meanwhile, gene transcriptions of VEGF-A, PDGF, and iNOS were up-regulated following with the increased amounts of FKN (from 1 to 10 ng/mL), with a dosage-dependent manner (Figure 2).

### 3.4 CX3CR1 interfering reduced the CX3CR1 expression

**Figure 3:**
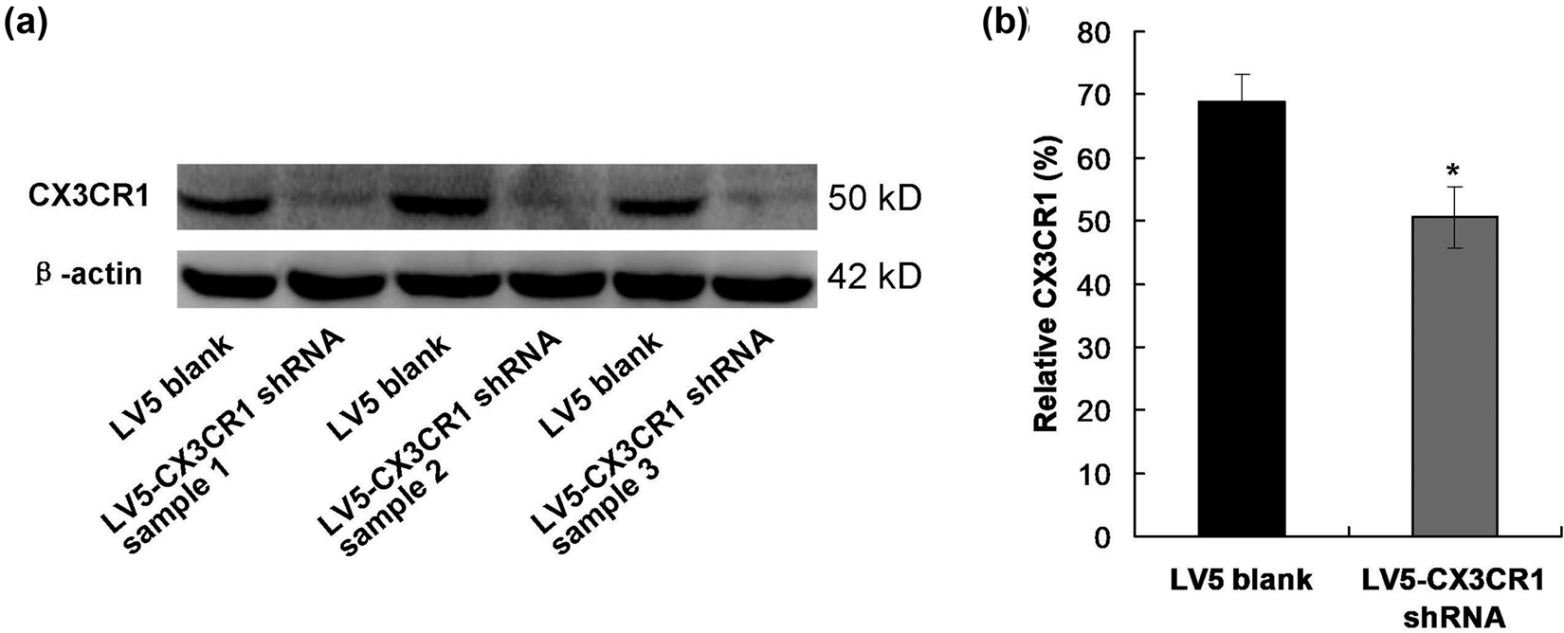
Determination for the interfering efficacy of CX3CR1 shRNA samples. (a) Western blot image. (b) Statistical for the relative expression of CX3CR1. CX3CR1: CX3C chemokine receptor 1.

In this study, we synthesized the interfering RNA, which was divided into three samples, including LV5-CX3CR1 shRNA sample 1, LV5-CX3CR1 shRNA sample 2, and LV5-CX3CR1 shRNA sample 3, all of which were loaded onto the SDS-PAGE and identified using western blot assay (Figure 3(a)). The results showed that all three samples significantly reduced the CX3CR1 expression in PVECs compared to those in LV5 blank group (Figure 3(b), *p* < 0.05). Meanwhile, there was no significant difference for CX3CR1 expression among the three samples (Figure 3(b)).

### 3.5 FKN activated p-Akt/Akt and p-Erk/Erk signaling pathway

**Figure 4:**
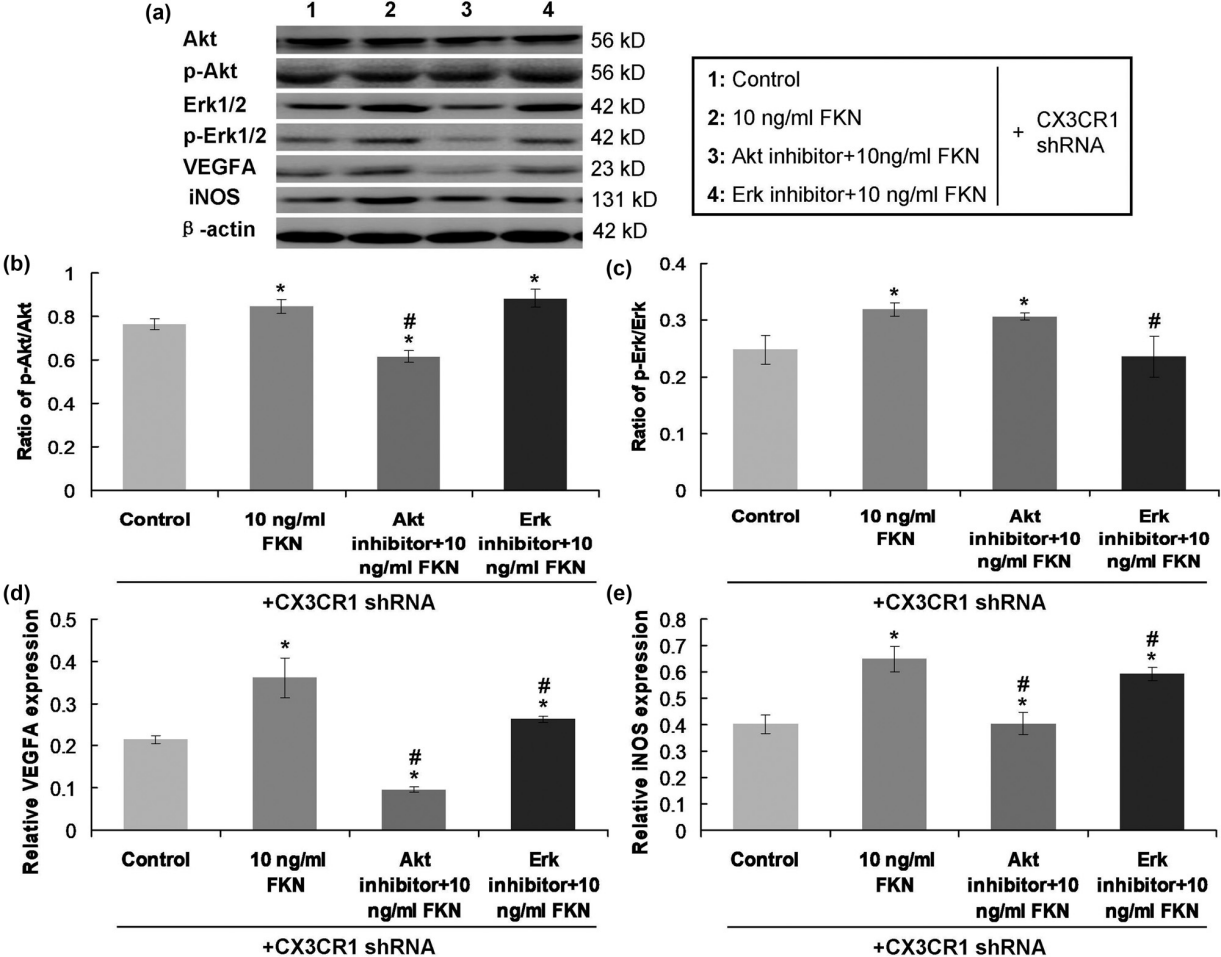
FKN and/or CX3CR1/Akt/Erk-inhibitor treatment on p-Akt/Akt ratio, p-Erk/Erk ratio, VEGF, and iNOS expression. (a) Western blot images for the expressions of molecules. (b) Comparison and analysis for ratios of p-Akt/Akt. (c) Comparison and analysis for ratios of p-Erk/Erk. (d) Comparison and analysis for expressions of VEGF molecule. (e) Comparison and analysis for expressions of iNOS molecule. *p < 0.05 versus Control group. ^#^ *p* < 0.05 versus 10 ng/mL FKN treatment. FKN: fractalkine, CX3CR1: CX3C chemokine receptor 1, Akt: protein kinase B, p-Akt: phosphorylated Akt, Erk1/2: extracellular regulated kinase1/2, p-Erk1/2: phosphorylated extracellular regulated kinase1/2, VEGFA: vascular endothelial growth factor A, iNOS: inducible nitric oxide synthase.

Due to the effects of FKN on the p-Akt and (p-Erk via CX3CR1 activation [15], the expressions of p-Akt and p-Erk were determined with western blot assay (Figure 4(a)). The findings indicated that the ratios of p-Akt/Akt (Figure 4(b)) and p-Erk/Erk (Figure 4(c)) in CX3CR1 shRNA-treated PVECs undergoing 10 ng/mL FKN treatment were significantly increased to those in PVECs in the Control group (*p* < 0.05). Moreover, comparing with the CX3CR1 shRNA-treated PVECs administered 10 ng/mL FKN, the ratio of p-Akt/Akt (Figure 4(b)) in CX3CR1 shRNA-treated PVECs administered Akt inhibitor and 10 ng/mL FKN was significantly decreased and ratio of p-Erk/Erk (Figure 4(c)) in CX3CR1 shRNA-treated PVECs administered Erk inhibitor and 10 ng/mL FKN was remarkably decreased (*p* < 0.05). However, there were no effects of Akt inhibitor + 10 ng/mL FKN on ratio of p-Erk/Erk and no effects of Erk inhibitor + 10 ng/mL FKN on ratio of p-Akt/Akt in the CX3CR1 shRNA-treated PVECs, compared to those in the 10 ng/mL FKN group. Therefore, FKN-activated p-Akt/Akt and p-Erk/Erk signaling pathway might be independent of the CX3CR1 molecule.

### 3.6 FKN increased VEGF-A and iNOS expression through activating Akt/Erk signaling pathway

**Figure 5:**
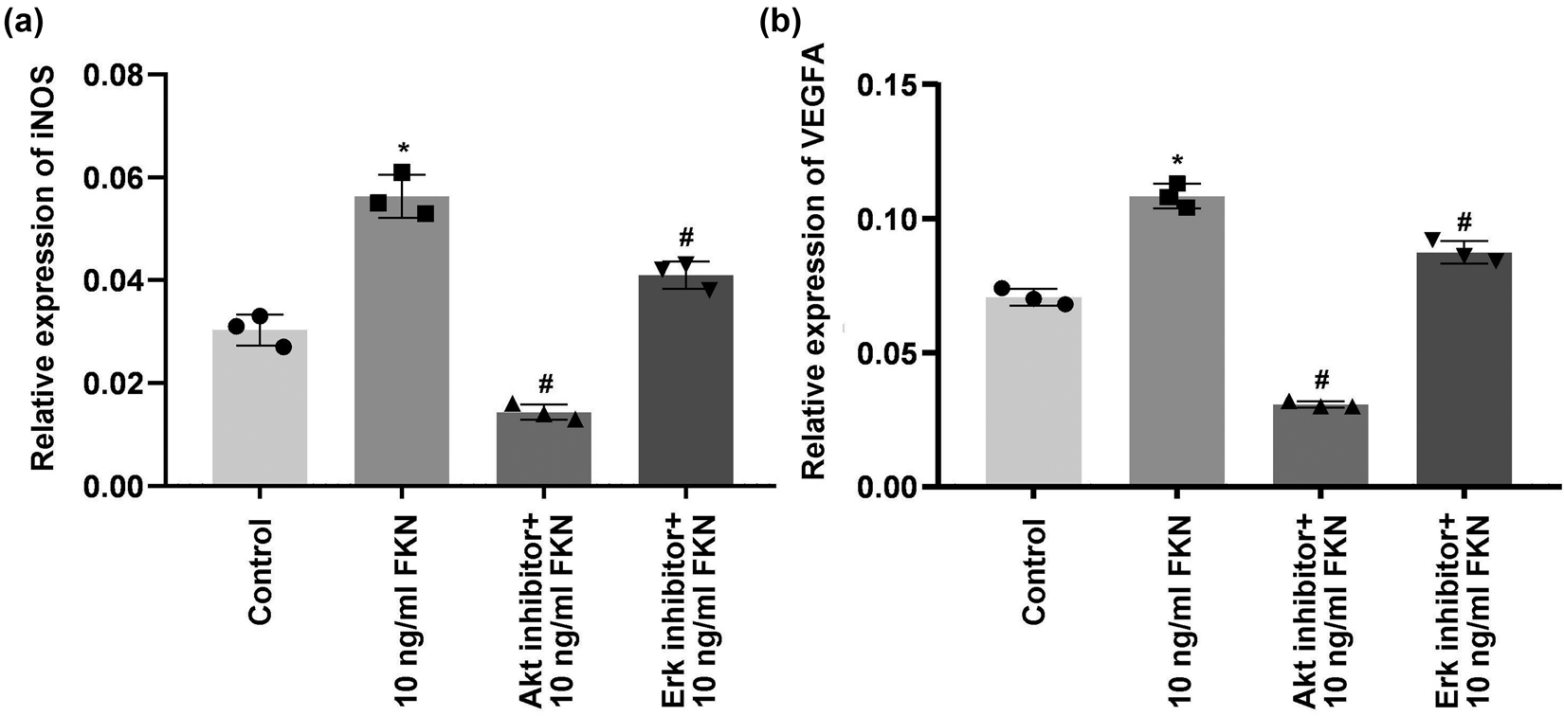
Reductive effects of 10 mg/mL FKN treatment combining Akt inhibitor or Erk inhibitor on gene transcriptions of iNOS and VEGF molecule. (a) RT-PCR analysis for gene transcription of iNOS. (b) RT-PCR analysis for gene transcription of VEGF. *p < 0.05 versus Control group. ^#^ *p* < 0.05 versus 10 ng/mL FKN treatment. FKN: fractalkine, Akt: protein kinase B, rk1/2: extracellular regulated kinase1/2, VEGFA: vascular endothelial growth factor A, iNOS: inducible nitric oxide synthase.

As shown in Figure 4(d) and (e), 10 ng/mL FKN significantly increased VEGF-1 and iNOS expression in CX3CR1 shRNA-treated PVECs compared to those in Control group (*p* < 0.05). Meanwhile, expressions of VEGF-1 (Figure 4(d)) and iNOS (Figure 4(e)) in CX3CR1 shRNA-treated PVECs were significantly decreased in Akt inhibitor + 10 ng/mL FKN group and Erk inhibitor + 10 ng/mL FKN group compared to those in 10 ng/mL FKN group (*p* < 0.05). Moreover, the RT-PCR assay findings also demonstrated that Akt inhibitor or Erk inhibitor administration markedly blocked the 10 ng/mL FKN-triggered gene transcription of iNOS (Figure 5(a)) and VEGF-A (Figure 5(b)) in PVECs. Therefore, the results suggest that FKN increased the VEGF-A and iNOS expression via activating the Akt/Erk signaling pathway.

### 3.7 FKN-treated macrophages enhanced activation of Akt/Erk signaling pathway

**Figure 6:**
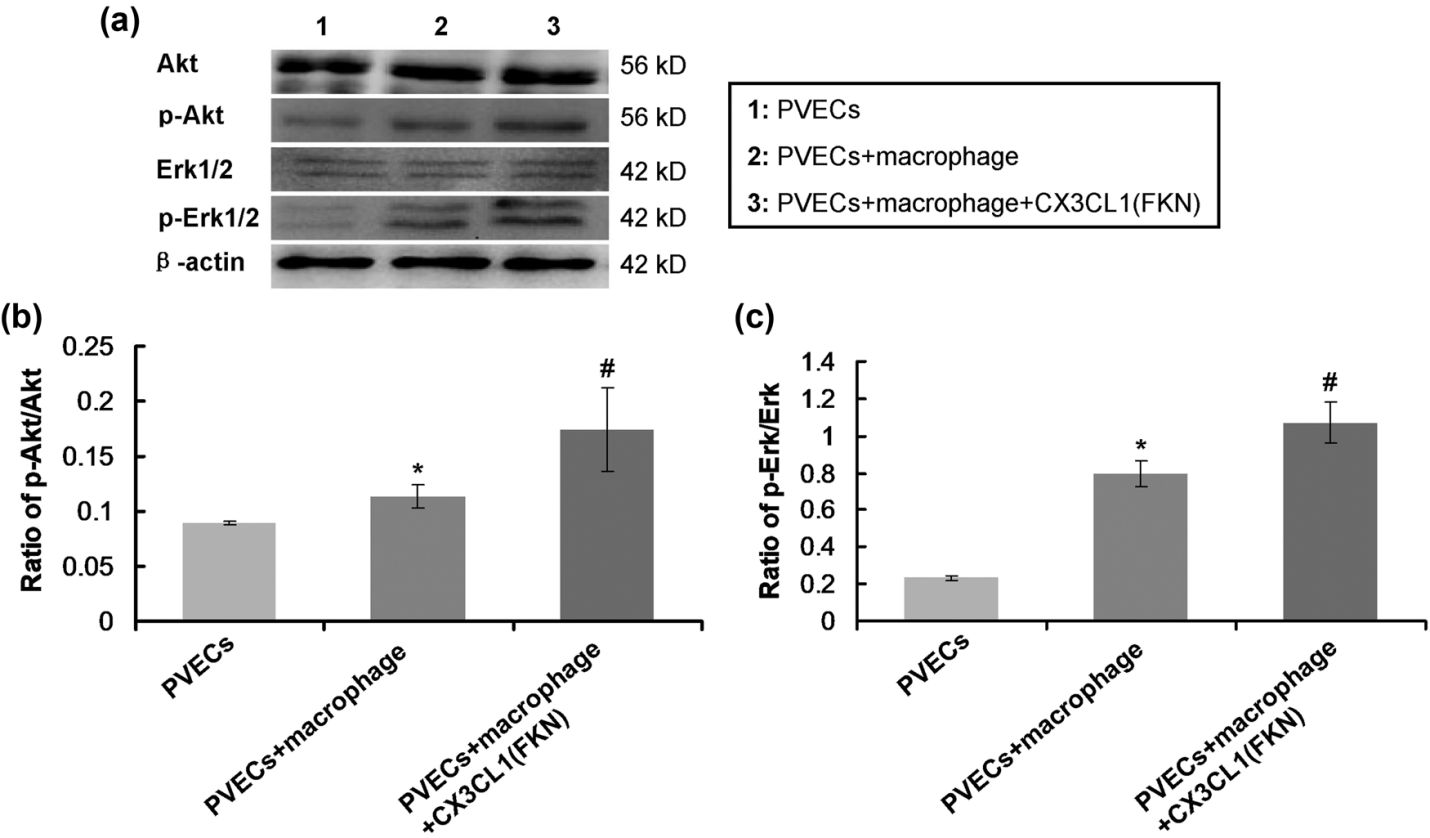
FKN-treated macrophages triggered the Akt/Erk signaling pathway in PVECs. (a) Western blot images for Akt/Erk signaling pathway associated molecules. (b) Analysis and comparison for ratio of p-Akt/Akt. (c) Analysis and comparison for ratio of p-Erk/Erk. *p < 0.05 versus PVECs group. ^#^ *p* < 0.05 versus PVECs + macrophage group. PVECs: pulmonary vascular endothelial cells, Akt: protein kinase B, p-Akt: phosphorylated Akt, E rk1/2: extracellular regulated kinase1/2, p-Erk1/2: phosphorylated extracellular regulated kinase1/2.

In order to clarify the effects of FKN-mediated secretion of macrophages on the Akt/Erk signaling pathway in PVECs, the western blot assay was carried out (Figure 6(a)). The results showed that FKN-treated macrophages (PVECs + macrophage + CX3CL1 group) remarkably activated the Akt signaling pathway (increased ratio of p-Akt/Akt) (Figure 6(b)) and Erk signaling pathway (increased ratio of p-Erk/Erk) (Figure 6(c)), compared to those in PVECs + macrophages group (*p* < 0.05). These results suggest that FKN might activate a molecule in macrophages, which then triggered the Akt/Erk signaling pathway in PVECs.

### 3.8 FKN-treated macrophages enhanced PDGF and VEGF-1 expression of PVECS

**Figure 7:**
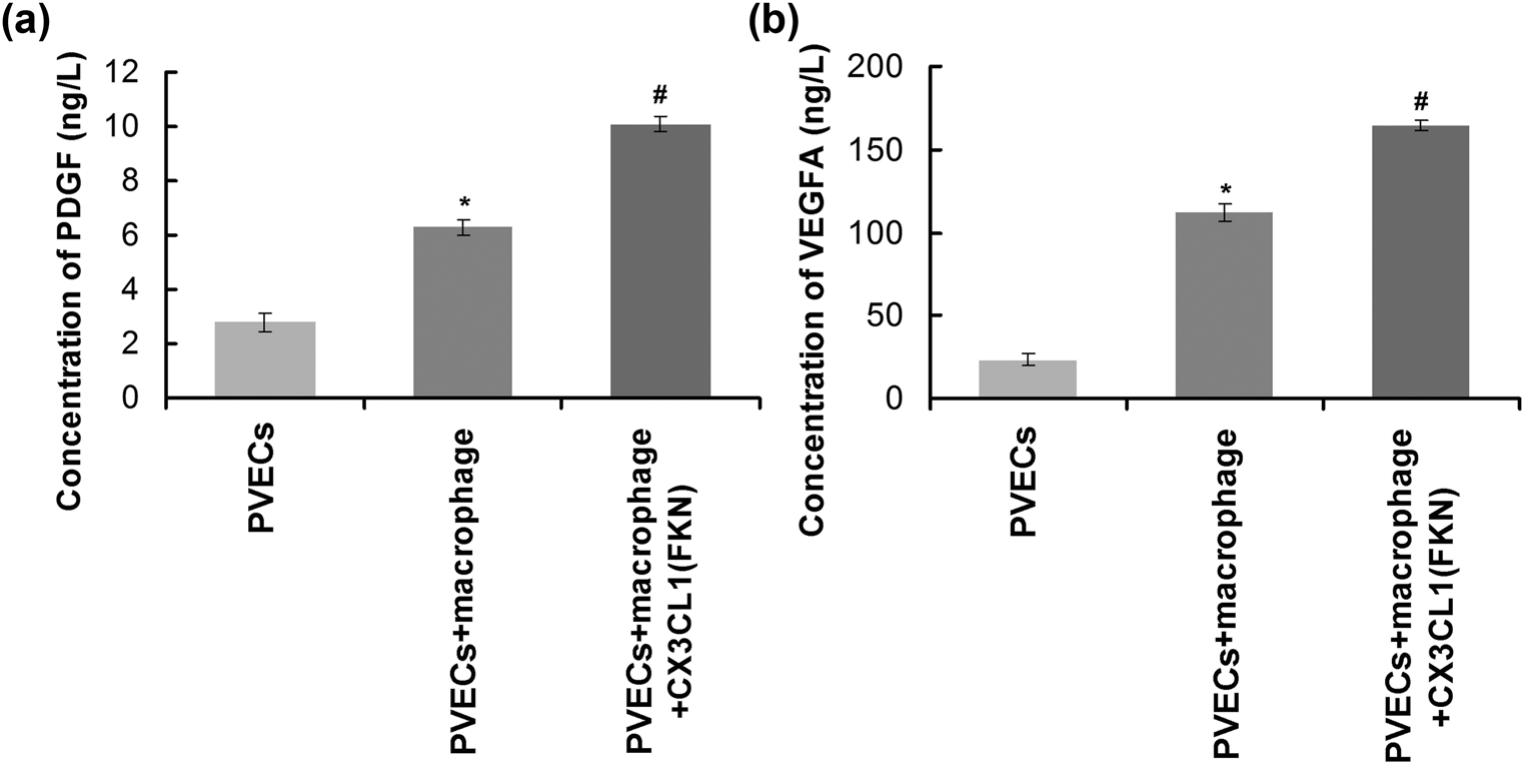
FKN-treated macrophages enhanced PDGF levels (a) and VEGF levels (b) in PVECs. *p < 0.05 versus PVECs group. ^#^ *p* < 0.05 versus PVECs + macrophage group. PVECs: pulmonary vascular endothelial cells, VEGFA: vascular endothelial growth factor A, PDGF: platelet-derived growth factor.

In order to evaluate the effects of FKN-treated macrophages on the angiogenic ability of PVECS, the angiogenesis-associated biomarkers, PDGF and VEGF-A, were examined using ELISA. The results indicated that FKN-treated macrophages significantly increased PDGF (Figure 7(a)) and VEGF-A (Figure 7(b)) levels in PVECs compared to those in the single PVECs (*p* < 0.05).

## 4 Discussion

HPS has been proven to be a progressive disorder that is characterized by worsening hypoxemia clinically [1]. According to the previous studies [6,7], FKN or CX3CL1 plays critical roles for initiating and developing the inflammation; therefore, CX3CL1 might be associated with the occurrence and progression of HPS. Therefore, this study determined the effects of FKN/CX3CL1 on pathogenesis of HPS in mouse PVECs and explored the underlying molecular pathological mechanisms.

In this study, we found that the FKN treatment (from 1 to 10 ng/mL FKN) is safe for the proliferation of PVECs according to the CCK-8 findings, which is consistent with the previous study described in the other cell line [16]. As well known, the tube formation is closely associated with the angiogenesis in the HPS; therefore, we determined the effects of FKN on the tube formation in PVECs. The findings showed that the PVECs exposed to FKN differentiated to form network of tube-like structures with a dosage-dependent manner, with the most obvious effect at a dosage of 10 ng/mL FKN.

Generally, the tube formation (angiogenesis) is correlated with the production of growth factors, such as VEGF-A, PDGF, and downstream angiogenic signaling mediator, iNOS [17]. The RT-PCR results demonstrated that gene transcriptions of VEGF-A, PDGF, and iNOS in FKN administered PVECs were markedly higher compared to those in PVECs in the Control group, with a dosage-dependent manner. The results suggest that FKN treatment enhanced VEGF-A, PDGF, and iNOS gene transcription. Therefore, the activation of VEGF-A, PDGF, and iNOS triggered the tube formation and finally induced the angiogenesis.

In this study, we also synthesized the interfering RNA targeting the CX3CR1, which demonstrated the obvious inhibitive effects on the expression of CX3CR1. Therefore, LV5-CX3CR1 shRNA was applied for the following experiments.

Because of the association between growth factors and Akt/Erk signaling pathways [18], the p-Akt and p-Erk were examined in the present study. The findings indicated that the ratio of p-Akt/Akt in CX3CR1 shRNA-treated PVECs administered Akt inhibitor and 10 ng/mL FKN was significantly decreased and ratio of p-Erk/Erk in CX3CR1 shRNA-treated PVECs administered Erk inhibitor and 10 ng/mL FKN was remarkably decreased, compared to CX3CR1 shRNA-treated PVECs administered 10 ng/mL FKN. These results suggest that FKN-activated p-Akt/Akt and p-Erk/Erk signaling pathway might be not directly associated with the CX3CR1 molecule. Different from our previously published study [19] reporting that CX3CR1 participating in the pulmonary angiogenesis by inhibiting Akt/Erk signaling pathway, our study further proved that FKN triggered the p-Akt/Akt and p-Erk/Erk signaling pathway.

Hou et al. [20] also reported that interaction between FKN and CX3CR1, however, is involved in the pathogenesis of endometriosis. Moreover, Gu et al. [19] reported that the Akt/Erk signaling pathway is correlated with the NO/NOS release and production. In our study, the expressions of VEGF-1 and iNOS in CX3CR1 shRNA-treated PVECs were significantly decreased in Akt inhibitor + 10 ng/mL FKN group and Erk inhibitor + 10 ng/mL FKN group compared to those in 10 ng/mL FKN group (*p* < 0.05). These results suggest that FKN increases VEGF-A and iNOS expression in CX3CR1 shRNA-treated PVECs via activating Akt/Erk signaling pathway. Therefore, FKN promoted VEGF-A/iNOS expression and triggered the p-Akt/Akt and p-Erk/Erk signaling pathway through modulating CX3CR1 molecule. However, Liu et al. [21] showed that CX3CR1 regulated angiogenesis and activation of p-ERK, iNOS, and VEGF in theHPS process, which is essentially consistent with the findings of this study.

Moreover, it is important to evaluate whether and how intravascular macrophages accumulate and mediate adhesion in the pulmonary vasculature as HPS develops [22]. In order to clarify the role of FKN in the intravascular macrophages accumulation HPS develops, this study investigated the effects of FKN on macrophages. The present study also demonstrated that FKN-treated macrophages significantly increased PDGF and levels in PVECs compared to those in the single PVECs. Thus, the macrophages accumulation in the progression of HPS was mediated by the activation of the FKN.

Although this received a few interesting results, there are also some limitations. First, the standardization of the inhibitor concentration on the cell lines has not been conducted. Therefore, this study cannot define whether these inhibitors can properly inhibit AKT and ERK phosphorylation. Second, though 30 min of incubation of inhibitors is enough due to our pre-experiments, a time-course experiment checking the level of phosphorylated proteins at various timepoints has not been carried out in this study. Third, the effect of individual Akt inhibitor or Erk inhibitor on the Akt phosphorylation or Erk phosphorylation has not been clarified. Fourth, this study did not show the results of similar experiments performed using either naive cells or cells treated with non-silencing (control) shRNA, which is the limitation of our study. In the following study, we would further confirm the results and conclusion of this study by conducting some associated experiments.

## 5 Conclusions

FKN promoted the tube formation in PVECs and triggered the pulmonary angiogenesis. The angiogenesis process was initiated through modulating the CX3CR1 molecule and growth factors (VEGF, PDGF, iNOS), and activating p-Akt/Akt and p-Erk/Erk signaling pathway. However, FKN-activated p-Akt/Akt and p-Erk/Erk signaling pathway might be independent the CX3CR1 molecule. Therefore, this study would provide the insight for application of CX3CR1 in modulating the angiogenesis. The link between FKN and the pulmonary angiogenesis in the PVECs might have the relationship to the HPS. Understanding the specific mechanism of the HPS might provide critical insight into the knowledges and treatments of human diseases. In the following study, we would explore the effects of FKN on the tube formation in the established animal model for further verifying the results of this study. Meanwhile, blocking the biological activity of the FKN or the related downstream molecules might affect the pathogenesis of HPS, which needs to be clarified in following study.
